# Hybrid text mining models for investigative keyword expansion on child sexual abuse in the dark web

**DOI:** 10.1371/journal.pone.0344470

**Published:** 2026-05-08

**Authors:** Jin Gyeong Kim, Jiyeon Kim

**Affiliations:** 1 Department of Computer and Information Engineering, Graduate School, Daegu University, Gyeongsan, South Korea; 2 Department of Computer Engineering, Daegu University, Gyeongsan, South Korea; Lorestan University, IRAN, ISLAMIC REPUBLIC OF

## Abstract

The distribution of child sexual abuse materials (CSAM) via the dark web continues to hinder digital investigations due to the network’s inherent anonymity and fragmentation. This work presents a comparative analysis of text mining techniques for extracting investigative keywords from CSAM-related content on the dark web and aims to establish a foundation for scalable, expandable keyword-based detection. Using a custom crawler, we collected data from 2,414 dark web pages indexed by the Torch search engine. Based on this dataset, three methods—TF-IDF, Eigenvector Centrality, and Word2Vec—were applied to extract CSAM-related keywords, and their effectiveness was evaluated through dark web search experiments measuring the retrieval performance of CSAM-related sites. Among the individual techniques, Eigenvector Centrality—a graph-based keyword ranking algorithm—showed the highest precision and contextual relevance by identifying structurally central terms within co-occurrence networks. Building on this, we developed hybrid models that combined Eigenvector Centrality with either TF-IDF or Word2Vec. In particular, the model integrating Eigenvector Centrality with Word2Vec-based semantic similarity proved most effective in expanding investigative clues and retrieving highly relevant keywords. Based on empirically collected and domain-specific dark web data, this work differs from prior studies by empirically demonstrating a multi-method approach that not only improves keyword accuracy but also enables the dynamic expansion of early-stage crime indicators. The proposed methodology offers practical value for automating the detection of illicit content and improving the operational efficiency of cyber investigations.

## Introduction

The dark web, which operates through encrypted communication protocols, has become a central medium for organized cybercrime activities such as digital sexual abuse, illegal gambling, and drug trafficking [1]. Unlike the surface web that is easily accessed via standard browsers like Chrome or Microsoft Edge, the dark web requires specialized software—most commonly the Tor browser—for access. Tor utilizes onion routing, a system that encrypts traffic and relays it through multiple nodes—entry, middle, and exit—thus concealing IP addresses and preserving user anonymity. This structural anonymity enables the dark web to serve as a platform for the dissemination of highly illegal and harmful content. In particular, child sexual abuse materials (CSAM) continue to circulate across hidden services with minimal regulatory oversight [2,3]. A well-known case is “Welcome to Video” in South Korea, where a large-scale CSAM distribution network operated by a single individual drew thousands of international users who accessed explicit content involving minors [4]. These crimes have evolved from informal exchanges between individuals into a profit-driven and organized ecosystem [5]. Operators deliberately dismantle and re-establish sites to avoid detection by law enforcement. This practice makes it increasingly difficult to track criminal activity or secure digital evidence. As CSAM production and distribution increasingly follow commercial and coordinated models, investigations require scalable, adaptive, and keyword-driven techniques to extract actionable leads from dynamic and opaque platforms [6]. However, most prior research has relied on static datasets extracted from previously known dark web forums or now-defunct sites, which often fail to reflect the latest patterns of criminal activity. The lack of real-time, domain-specific data collection frameworks has limited the ability to detect newly emerging threats and expand investigative leads. While existing studies have applied machine learning and content classification techniques to analyze dark web data, most have primarily focused on surface-level patterns or isolated terms. Few have addressed the need for methods that can dynamically expand early investigative clues into deeper leads across platforms. To address these limitations, we propose a text mining–based approach for the automated collection and analysis of textual data from dark web sources distributing CSAM. Specifically, we compare three representative techniques—TF-IDF, Eigenvector Centrality, and Word2Vec—to evaluate their effectiveness in extracting investigative keywords. Furthermore, we design combined models that improve contextual relevance and detection accuracy by integrating the structural centrality captured by Eigenvector Centrality with either the statistical rarity captured by TF-IDF or the semantic similarity provided by Word2Vec. The remainder of this paper is structured as follows.

The related works review existing studies on dark web investigation and keyword extraction using text mining.

The materials and methods section then describes the dataset construction process and the individual text mining models. The results section presents the proposed combined models, compares their performance, and evaluates their effectiveness in identifying CSAM-related content. The discussion examines the robustness and generalizability of the proposed approach, including additional experiments using alternative seed keywords, and the conclusion summarizes the key findings and outlines directions for future research.

## Related works

### Research on dark web investigation trends

The dark web has emerged as a central medium for illegal activities such as drug trafficking, unlawful gambling, and the distribution of CSAM. To respond to these crimes, various investigative strategies have been introduced to detect and monitor criminal activities across dark web platforms. Machine learning approaches have been widely applied to classify and analyze content from the dark web. One line of research involved extracting HTML tags and textual content from dark web pages and applying neural networks in combination with semi-supervised support vector machines to classify crime types [7]. Another approach employed convolutional neural networks (CNNs) and KeyBERT to identify relevant keywords and classify images and texts extracted from illicit websites [8]. Deep learning methods, such as long short-term memory (LSTM) models, have also been trained on dark web forum data to detect criminal behaviors [9], while transductive semi-supervised learning techniques have been adopted for improved detection using domain-specific datasets [10]. Beyond content classification, structural and network-based methods have been used to identify patterns in criminal ecosystems. Studies have analyzed hyperlink networks among dark web sites to uncover the evolution of criminal groups and identify high-centrality nodes [11,12]. Other research focused on financial traces by analyzing Bitcoin transactions—commonly used on the dark web—to estimate the scale of illegal marketplaces and map user behaviors [13]. These combined efforts reflect a growing trend toward integrating content analysis, network structure, and transaction-level data to better expose and understand hidden criminal infrastructures.

However, most of these studies have relied on static or outdated datasets, often based on previously identified or now-defunct illicit sites. As the dark web evolves rapidly and many such platforms are taken offline or replaced, these approaches struggle to reflect the current threat landscape. More importantly, there remains a lack of research on establishing real-time collection systems that can accurately identify crime-relevant pages and dynamically expand early investigative clues into broader leads. To address these limitations, this work develops a custom crawler to collect live dark web data and proposes a text mining–based framework that can extract and scale high-precision investigative keywords for active cybercrime detection.

### Cyber investigation research based on text mining

Text mining has become a fundamental methodology in cybercrime analysis, enabling researchers to extract meaningful patterns from large-scale unstructured data. Prior studies have demonstrated that text-mining techniques can support security and risk analytics across various application domains [14]. In the crime analysis field, graph-based community detection and clustering algorithms—such as the Louvain method—have been applied to real-world crime reports to uncover threat-related structures and derive investigative cues [15]. WordNet-based semantic analysis has also been employed to identify relationships among crime-related terminology and support cybercrime investigation workflows [16]. In addition, machine-learning models built on TF–IDF representations have been used to classify crime-related textual records from public datasets, including categorizing theft or other offense types using XGBoost and similar classifiers [17]. Within dark web research specifically, machine-learning approaches have been applied to classify cybercrime offenses and analyze linguistic characteristics of illicit online communications, demonstrating the utility of embedding-based and feature-based text representations [18]. Graph-embedding techniques have further been introduced to track the evolution of hacker-forum terminology over time and proactively identify emerging cyber threats [19]. Recent dark web research has widely applied text-mining and machine-learning techniques to analyze illicit content, using TF–IDF and related term-weighting schemes as standard baselines for representing salient terms, and combining them with methods such as K-means clustering, decision trees, logistic regression, and N-gram-based topic modeling to categorize onion sites and characterize dark web forums [20–23]. From a network analysis standpoint, graph-based metrics such as degree distribution, centrality, and PageRank have been applied to identify influential sites and trace content distribution paths in dark web ecosystems [24–27], providing insights into the structure and flow of illicit information,

While these studies have advanced both content-based and structure-aware approaches, many studies still rely on single techniques and have not been empirically validated in real-world investigations. Moreover, few approaches focus on building keyword-driven detection models that can rapidly identify early-stage criminal clues and actively expand them into broader investigative leads. To address these limitations, we compare multiple text mining techniques for extracting CSAM-related keywords and evaluate their effectiveness in dark web environments. In addition to individual methods, we propose hybrid models designed to improve both the precision and scalability of investigative keyword extraction, with an emphasis on applicability to actual forensic workflows.

## Materials and methods

### Keyword extraction with individual text mining models

This section describes the process of extracting CSAM-related keywords from dark web sources. Using a seed keyword, we developed a crawler to collect CSAM-related pages from the dark web. Based on the collected dataset, we applied three text mining techniques—TF-IDF, Eigenvector Centrality, and Word2Vec—to extract key terms. We then conducted a comparative evaluation of these models to identify the most effective approach for detecting high-relevance CSAM keywords.

#### Data collection from the dark web.

**Fig 1:**
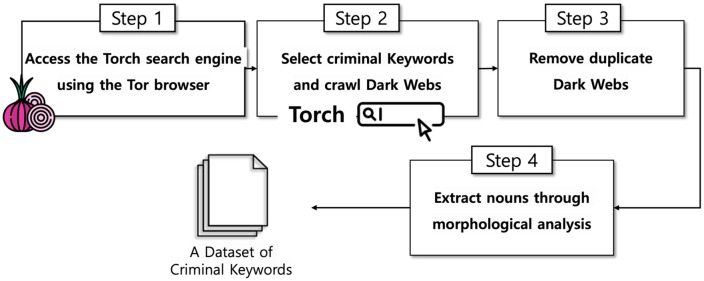
Workflow of the dark web keyword crawler for crime data collection.

To construct a dataset for CSAM-related keyword analysis, a dark web crawler was developed using the Torch search engine. Torch is one of the longest-running search engines on the Tor network and provides access to a wide range of.onion domains [28]. Fig 1 illustrates the overall keyword crawling process.

In the first step, the crawler establishes a session through the Tor browser and accesses Torch. Next, it enters seed keywords—terms known to be associated with CSAM—into the search engine. In this study, the term “Lolita”, which signifies an adult man’s sexual attraction to young girls, was selected as a seed keyword. Using this term, the crawler retrieved 2,414 dark web pages indexed by Torch that were potentially related to child sexual abuse. The crawler then follows each resulting link and collects all visible text data from the retrieved pages.

Throughout this process, the crawler was employed solely for academic research purposes, and the collected raw data were neither publicly released nor redistributed. We utilized textual data collected from the Torch search engine, accessed via the Tor browser, using CSAM-related seed keywords as search inputs. The resulting dataset consists exclusively of textual content from dark web sites retrieved with the designated seed keywords; no illegal CSAM content such as images or videos was downloaded or stored.

To prevent redundancy in the corpus, previously collected content is compared against new entries and duplicates are removed. The collected raw text corpus then undergoes a multi-stage preprocessing procedure. First, unnecessary text components such as HTML tags, scripts, and boilerplate markup are removed. The remaining data are processed through a regular expression–based tokenizer and morphological analysis to extract tokens, with a particular focus on nouns that are more likely to represent core entities or concepts. Standard English stopwords and additional stopwords defined to reflect the characteristics of the dark web domain are removed to reduce noise caused by meaningless or non-informative text. In addition, random strings, system placeholders, and markup artifacts are manually excluded. All subsequent analyses are performed on this refined set of textual tokens, which constitutes a de-duplicated, text-only dataset derived from CSAM-related dark web pages.

The reason this study adopted an English-based analysis is that nearly all dark web CSAM-related sites collected were written in English. In our crawling results, excluding inaccessible pages, 100% of the retrieved sites were English-based. Accordingly, focusing on English for keyword extraction and model construction was deemed the most appropriate approach. At the same time, the TF-IDF, Eigenvector Centrality, and Word2Vec techniques used in this study are language-agnostic; with appropriate adjustments to morphological structures and tokenization rules, they can be applied to other languages as well.

Building on this dataset, the study proposes a text mining–based approach for the automatic collection and analysis of textual data from dark web sources associated with the distribution of CSAM. Three representative techniques are applied: term frequency–inverse document frequency (TF–IDF), which quantifies the importance of terms based on document-level frequency; eigenvector centrality, which identifies structurally central keywords within a term co-occurrence network; and Word2Vec, which captures semantic similarity between terms. The effectiveness of crime-related keywords extracted by each individual method is first validated, and the performance of TF–IDF, eigenvector centrality, and Word2Vec in investigative keyword extraction is then compared and evaluated. Based on these experiments, a hybrid model is designed that integrates statistical rarity information from TF–IDF and semantic similarity information from Word2Vec, centered on eigenvector centrality, with the aim of simultaneously improving the contextual relevance and detection accuracy of extracted keywords. The study qualitatively and quantitatively compares the performance of the individual and hybrid models to evaluate their effectiveness in detecting CSAM-related content.

The data collection and analysis procedures were designed to comply with all applicable legal and ethical standards, including regulations concerning CSAM. Although the raw data used in this research may contain textual expressions related to CSAM collected from the dark web, these data were used exclusively for internal analysis within the research team and were not shared externally in any form. The dataset employed in this paper consists of derivative text-only data generated from the raw corpus, and it does not include any multimedia files such as images or videos. Moreover, the crawler was implemented as a strict text-only collector and did not download any images, videos, or file attachments from target pages, thereby minimizing legal and ethical risks and preventing researchers from being directly exposed to illegal materials.

#### Ethical and legal compliance.

All data collection and analysis procedures were conducted in accordance with applicable legal and ethical standards for research related to CSAM. In this study, only textual data were collected and analyzed, and no illegal CSAM content, such as images or videos, was downloaded, stored, or viewed by the researchers. The crawler was implemented as a text-only collector, so multimedia files were not collected. All raw corpus data were stored in a secure local research environment, with access restricted to authorized members of the research team for research purposes only. Furthermore, because the methodology proposed in this study relies exclusively on derived, text-based analytical outputs, it does not enable readers to access CSAM content.

#### Terms and conditions compliance.

Data collection and analysis were conducted in accordance with the publicly available terms and conditions and acceptable-use policies of the data sources used in this study (including the Tor Browser software and the Torch search service used to locate.onion pages). The crawler accessed only content retrievable through standard browsing without authentication, did not circumvent access controls, and did not redistribute third-party page content.

#### Text mining techniques for CSAM keyword extraction.

This section introduces three distinct text mining methods—TF-IDF, Eigenvector Centrality, and Word2Vec—used to extract keywords related to CSAM from dark web text data. Each method is described in terms of its conceptual framework and analytical strengths. Their comparative performance and validation results are addressed in a subsequent section.

**TF-IDF-based keyword extraction:** TF-IDF is a statistical method used to evaluate how important a word is within a document relative to a collection of documents. It favors terms that appear frequently in a specific document but are rare across the broader corpus, making it effective for extracting distinctive terms [29–32]. When applied to the collected dataset of 71,666 nouns from 2,414 dark web pages, TF-IDF surfaced a set of top-ranked keywords that reflect the underlying structure and distribution mechanisms of CSAM-related content. These keywords were ranked by their TF-IDF scores, which quantify a term’s distinctiveness by combining its frequency within a document and its rarity across the corpus. Table 1 summarizes the extracted keywords.

**Table 1:** Top 20 keywords extracted using TF-IDF.

No	Keyword	Score	No	Keyword	Score
**1**	maxchan	0.555582	**11**	child	0.006743
**2**	mixedlolitas	0.278278	**12**	erotic	0.005986
**3**	countries	0.123877	**13**	community	0.005578
**4**	stronghold	0.034613	**14**	hanamaru	0.004423
**5**	illegal	0.023052	**15**	sonya	0.004365
**6**	city	0.017943	**16**	camera	0.004074
**7**	language	0.015146	**17**	collection	0.00347
**8**	pornoslonik	0.009811	**18**	videos	0.003407
**9**	gallery	0.008874	**19**	illusion	0.003136
**10**	endchan	0.007779	**20**	zenphoto	0.002985

The highest-ranking keyword, “maxchan” (1st), refers to publicly available login credentials on a dark web site, which are used to facilitate repeated user access. “mixedlolitas” (2nd) appears as a hyperlink embedded within site navigation, while “zenphoto” (20th) denotes software used to organize and present multimedia files on CSAM-sharing sites. “pornoslonik” (8th) and “camera” (16th) are related to tools used for producing or storing exploitative materials. Community structures are reflected in terms like “endchan” (10th) and “community” (13th), which suggest the presence of forums or boards for sharing materials and peer communication. Personal identifiers such as “hanamaru” (14th) and “sonya” (15th) likely function as usernames or pseudonyms associated with content creators or distributors. In addition, broader contextual terms—”countries” (3rd), “city” (6th), and “language” (7th)—highlight the cross-national and multilingual nature of these platforms, reflecting the global reach of CSAM dissemination. Although many of these keywords do not explicitly describe abusive content, they provide investigative value by revealing the infrastructural, communicative, and operational layers of dark web ecosystems.

**Eigenvector centrality-based keyword extraction:** Eigenvector Centrality is a graph-based ranking algorithm that evaluates a keyword’s importance based on both the number and influence of its co-occurring neighbors. This allows it to identify structurally central terms in a co-occurrence network, often capturing deeply embedded or thematically cohesive concepts [32–35].

In this study, Eigenvector Centrality identified high-impact CSAM-related keywords such as “child” (1st), “zoo” (3rd), “pedomoms” (4th), and “jblinks” (6th), which showed strong semantic relevance to exploitative content and related community structures. These terms occupy structurally central positions within the co-occurrence network, indicating their frequent appearance alongside many other key terms. Table 2 presents the top 20 keywords ranked by their eigenvector centrality scores.

**Table 2:** Top 20 keywords extracted using eigenvector centrality.

No	Keyword	Score	No	Keyword	Score
**1**	child	0.306826	**11**	little	0.165413
**2**	lola	0.238443	**12**	cute	0.153748
**3**	zoo	0.234528	**13**	pic	0.153341
**4**	pedomoms	0.232663	**14**	pictures	0.149286
**5**	toddlers	0.232663	**15**	model	0.147735
**6**	jblinks	0.232663	**16**	nude	0.146937
**7**	kitty	0.232663	**17**	young	0.133342
**8**	myloveboard	0.232663	**18**	photo	0.12481
**9**	thumbnailed	0.228013	**19**	image	0.124321
**10**	videos	0.187591	**20**	beautiful	0.117419

“child” (1st) had the highest centrality, reaffirming the primary focus of these networks on minors. “lola” (2nd), a stylized variant of “Lolita”, and “jblinks” (6th) function as hyperlink labels or navigational anchors connecting users across multiple sites. “Myloveboard” (8th), a known category within the “MixedLolitas” ecosystem, likely serves as a thematic hub organizing exploitative content. Other keywords point to specific behaviors or victim profiles.

For instance, “zoo” (3rd) and “pedomoms” (4th) reference extreme themes such as animal exploitation or parental abuse. “Toddlers” (5th) indicates an even narrower target age group, likely children aged 1–3. Media-related keywords such as “thumbnailed” (9th), “videos” (10th), “pic” (13th), “pictures” (14th), “photo” (18th), and “image” (19th) highlight practices of visual content organization and dissemination. Terms like “little” (11th), “cute” (12th), and “young” (17th) reflect grooming language or descriptors that may serve to attract specific audiences. The appearance of terms such as “nude” (16th), “model” (15th), and “beautiful” (20th) suggest attempts to normalize or stylize illegal content.

Overall, this analysis highlights that CSAM content on the dark web is both semantically rich and structurally organized around central keywords performing navigational and thematic roles. The results demonstrate Eigenvector Centrality’s strength in identifying deeply embedded keywords that define the topology of exploitative content networks, thereby providing practical insights for investigative detection and intervention.

**Word2Vec-based keyword extraction:** Word2Vec is a neural embedding model that represents words in a continuous vector space, allowing for the identification of semantically similar or related terms. It is particularly adept at surfacing slang or euphemistic expressions that might not be captured by frequency-based models [36–39]. Table 3 presents the top 20 keywords extracted using the Word2Vec technique from the dark web CSAM dataset. The similarity scores indicate the semantic proximity of each keyword to the target context within the embedding space.

**Table 3:** Top 20 keywords extracted using Word2Vec.

No	Keyword	Score	No	Keyword	Score
**1**	guys	0.998555	**11**	huge	0.998134
**2**	boys	0.99849	**12**	hard	0.998131
**3**	sweet	0.998361	**13**	russian	0.998117
**4**	models	0.998346	**14**	bad	0.998068
**5**	pussy	0.998274	**15**	loves	0.998032
**6**	ass	0.998262	**16**	enjoy	0.998023
**7**	cutie	0.998249	**17**	face	0.998005
**8**	remember	0.998246	**18**	anybody	0.997985
**9**	school	0.998199	**19**	darkweb	0.997958
**10**	telegru	0.998169	**20**	american	0.997952

The highest-ranked keywords, such as “guys” (1st) and “boys” (2nd), reflect the strong association with the thematic core of CSAM, especially targeting young male victims. Emotionally expressive terms like “sweet” (3rd), “cutie” (7th), and “enjoy” (16th) suggest promotional or evaluative language often used within exploitative communities.

Sexually explicit slang terms are also prominent, including “pussy” (5th) and “ass” (6th), which are frequently used to describe body parts in a sexualized context. Notably, “telegru” (10th) appears as a slang variation of “Telegram,” indicating platforms commonly mentioned in dark web communications. Geographic indicators such as “Russian” (13th) and “american” (20th) imply the global distribution of such content, often referenced through national or regional tags. The term “school” (9th) anchors the content in specific social contexts, while “models” (4th) may reflect attempts to legitimize or disguise exploitative imagery using softened terminology. These findings highlight the model’s ability to extract not only semantically related keywords but also criminal slang, emotional rhetoric, platform references, and distributional clues. Word2Vec proves effective for uncovering concealed linguistic signals within CSAM-related content on the dark web, offering valuable insights for interpreting criminal lexicons and designing robust detection strategies.

#### Comparative evaluation of individual models.

**Fig 2:**
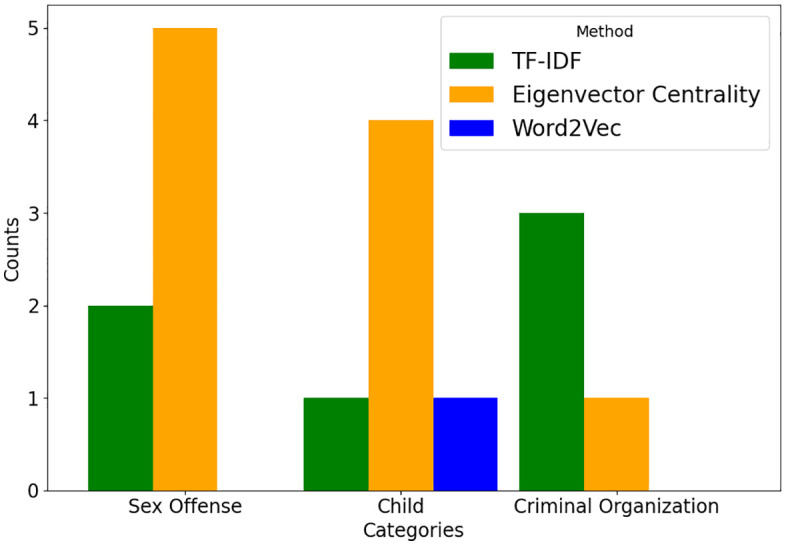
Categorization of the top 20 keywords extracted using individual text mining techniques by crime-related category.

To assess the performance of the three techniques, we conducted a comparative analysis based on two metrics: keyword categorization and retrieval accuracy. Fig 2 categorizes the top 20 keywords from each model into three CSAM-relevant categories: sexual crimes, children, and criminal organizations.

Eigenvector Centrality yielded the most balanced results, identifying five keywords related to sexual crimes (e.g., “lola”, “pedomoms”, “jblinks”), four to children (e.g., “child”, “toddlers”), and one to criminal organizations (“myloveboard”). This balance indicates that the graph-based mechanism of Eigenvector Centrality effectively captures structurally important and thematically meaningful keywords embedded in dark web text.

TF-IDF extracted two keywords associated with sexual crimes (“pornoslonik”, “erotic”), one with children (“child”), and three with criminal organizations (“maxchan”, “mixedlolitas”, “endchan”). While TF-IDF performs well in identifying terms with strong document-level frequency characteristics, some extracted keywords may reflect infrastructural elements or general web terminology rather than direct criminal indicators.

In contrast, Word2Vec’s results skewed toward emotionally expressive or colloquial language (e.g., “sweet”, “cutie”, “enjoy”), with only one keyword (“boys”) clearly categorized under child-related terms. This pattern demonstrates Word2Vec’s strength in capturing semantic similarity rather than domain-specific criminal terminology, resulting in broader yet occasionally less precise keyword groups.

The CSAM site identification accuracy used in this study represents the proportion of sites identified as CSAM-related among the valid sites collected from the dark web using a specific keyword *k*, and is defined as shown in Equation (1).


$$\begin{array}{c} Accuracy=\frac{C_{k}}{N_{k}}\times \text{1}\text{0}\text{0} \end{array}$$ (1)

In Equation (1), $N_{k}$ denotes the number of valid sites collected using keyword *k* that were accessible and whose actual content could be confirmed, while $C_{k}$ represents the number of those valid sites classified as CSAM-related. Sites that were offline, returned error codes, or were otherwise inaccessible were excluded from the analysis, and accuracy was computed solely based on verifiable, valid sites.

To obtain these measurements, major keywords were submitted to the dark web search engine Torch, and the sites exposed on the result pages were collected. Each collected site was then manually inspected in full to determine whether it was related to CSAM. CSAM-related sites were further categorized into two types: (1) Child Sexual Abuse Material Distribution, referring to sites that directly facilitate the upload or download of child sexual abuse material, and (2) Sexual Abuse Community, referring to sites that primarily share news, information, or discussions related to sexual crimes involving minors. Site classification was conducted manually by the researchers according to predefined criteria. Each site was evaluated based on whether it directly distributed CSAM content or exhibited characteristics of a CSAM-related community. Due to the sensitive nature of the data and ethical considerations, independent multi-annotator labeling and the assessment of inter-rater reliability were not performed. Instead, classification consistency was ensured by applying clearly defined annotation guidelines and conducting an exhaustive review of all collected sites under the same criteria. The validation results obtained using this procedure are presented in Table 4.

**Table 4:** Validation accuracy based on keywords extracted from individual models.

Model	No	Keywords	Types of crime occurrences		Accuracy (%)
			(1) Child sexual abuse material distribution (%)	(2) Sexual abuse Community (%)	
			Number of Identified Sites (%)		
**TF-IDF**	1	maxchan	0 (0)	0 (0)	0
	2	mixedlolitas	0 (0)	0 (0)	0
	3	countries	2,106 (15.88)	7,476 (56.37)	72.25
	4	stronghold	6 (0.16)	201 (5.2)	5.36
	5	illegal	174 (1.66)	3,172 (30.21)	31.87
**Eigenvector Centrality**	1	child	1,964 (21.12)	5,445 (58.54)	79.66
	2	lola	1,265 (62.13)	496 (24.36)	86.49
	3	zoo	1,312 (59.29)	668 (30.19)	59.48
	4	pedomoms	1,267 (97.54)	0 (0)	97.54
	5	toddlers	1,284 (81.37)	225 (14.26)	95.63
**Word2Vec**	1	guys	1,851 (13.92)	3,692 (27.77)	41.69
	2	boys	1,851 (8.33)	17,948 (80.75)	89.08
	3	sweet	3,692 (37.58)	5,170 (52.63)	90.21
	4	models	3,212 (16.13)	10,652 (53.49)	69.62
	5	pussy	404 (43.44)	448 (48.17)	91.61

Keywords derived based on Eigenvector Centrality demonstrated consistently high retrieval accuracy for CSAM-related sites, with four out of five keywords achieving over 79% and some exceeding 95% (“pedomoms” at 97.54%, “toddlers” at 95.63%). These results affirm the method’s ability to uncover high-impact, structurally central terms. In contrast, TF-IDF’s top five keywords showed wide variability in accuracy. Three keywords (“maxchan”, “mixedlolitas”, and “stronghold”) scored below 6%, whereas only ‘countries’ achieved a relatively high accuracy of 72.25%. This variance indicates TF-IDF’s sensitivity to context and its tendency to extract general or infrastructural terms when applied to diverse web content. Word2Vec displayed intermediate performance, with several keywords exceeding 80% accuracy (“boys” at 89.08%, “sweet” at 90.21%, “pussy” at 91.61%), yet others showed semantic drift, reducing their forensic utility.

These findings indicate that Eigenvector Centrality is the most effective individual method for extracting structurally important CSAM-related keywords within the dark web ecosystem. Its awareness of network topology enables the identification of deeply embedded terms that are frequently central to illicit content clusters. However, despite its strength in capturing co-occurrence-based centrality, this approach may overlook context-specific or semantically nuanced clues that are equally critical in digital investigations. Therefore, the following section refines the approach by introducing hybrid models that integrate complementary methods to overcome these limitations and enhance the practical applicability of keyword-based crime detection.

### Refinement of investigative keywords using combined text mining models

Building on the findings of the earlier analysis, which highlighted the relative strengths and weaknesses of TF-IDF, Eigenvector Centrality, and Word2Vec, this section aims to enhance keyword extraction performance by combining their complementary advantages. While Eigenvector Centrality proved effective in identifying structurally central terms within co-occurrence networks, its limitations in capturing context-specific and semantically rich expressions necessitate a more holistic approach.

To this end, we introduce four hybrid models that integrate Eigenvector Centrality with either TF-IDF or Word2Vec. These combined models are designed to balance structural importance with semantic relevance, thereby improving the contextual accuracy and investigative utility of extracted CSAM-related keywords. Specifically, Combined Models 1 and 2 integrate Eigenvector Centrality with TF-IDF, while Combined Models 3 and 4 incorporate Word2Vec-based semantic similarity to expand the range of crime-relevant terms.

#### Combined Models 1 and 2: Eigenvector centrality–TF-IDF based models.

In Combined Model 1, the top 20 keywords extracted via Eigenvector Centrality were selected based on their structural centrality within the co-occurrence network. These were paired with the top 20 TF-IDF keywords, excluding 2 overlapping terms, resulting in 18 unique TF-IDF keywords. A total of 360 keyword pairs (20 × 18) were constructed to combine structurally prominent and statistically rare terms, enhancing the discovery of crime-related indicators. In Combined Model 2, the same 20 Eigenvector Centrality keywords were combined with a broader set of 6,974 TF-IDF keywords. After removing 19 overlapping terms, 6,955 non-redundant TF-IDF keywords were retained, producing 139,100 unique keyword pairs (20 × 6,955). To ensure consistent scoring across differing value scales, Min-Max normalization was applied, transforming all keyword scores into a [0, 1] range. Subsequently, the final score for each keyword pair was computed by summing the normalized Eigenvector Centrality value $x_{i}$ of keyword *i* and the normalized TF-IDF value $y_{t}$ of keyword *t*, as shown in Equation (2). This scoring mechanism integrates both structural centrality and statistical rarity to highlight investigative relevance. The top 20 keyword pairs generated from Combined Models 1 and 2 are presented in Table 5.

**Table 5:** Top 20 keyword pairs extracted from combined models using eigenvector centrality and TF-IDF.

No	Combined Model 1		Score(i,t)	No	Combined Model 2		Score(i,t)
	Keyword Pairs				Keyword Pairs		
**1**	child	maxchan	2	**1**	child	maxchan	2
**2**	lola	maxchan	1.63896	**2**	lola	maxchan	1.63896
**3**	zoo	maxchan	1.618295	**3**	zoo	maxchan	1.618295
**4**	pedomoms	maxchan	1.608444	**4**	pedomoms	maxchan	1.608444
	toddlers	maxchan	1.608444		toddlers	maxchan	1.608444
	jblinks	maxchan	1.608444		jblinks	maxchan	1.608444
	kitty	maxchan	1.608444		kitty	maxchan	1.608444
	myloveboard	maxchan	1.608444		myloveboard	maxchan	1.608444
**5**	thumbnailed	maxchan	1.583897	**5**	thumbnailed	maxchan	1.583897
**6**	child	mixedlolitas	1.49818	**6**	child	mixedlolitas	1.500874
**7**	videos	maxchan	1.370481	**7**	videos	maxchan	1.370481
**8**	little	maxchan	1.253391	**8**	little	maxchan	1.253391
**9**	child	countries	1.21877	**9**	child	countries	1.222964
**10**	cute	maxchan	1.191803	**10**	cute	maxchan	1.191803
**11**	pic	maxchan	1.189653	**11**	pic	maxchan	1.189653
**12**	pictures	maxchan	1.168245	**12**	pictures	maxchan	1.168245
**13**	model	maxchan	1.160054	**13**	model	maxchan	1.160054
**14**	nude	maxchan	1.155841	**14**	nude	maxchan	1.155841
**15**	lola	mixedlolitas	1.13714	**15**	lola	mixedlolitas	1.139834
**16**	zoo	mixedlolitas	1.116475	**16**	zoo	mixedlolitas	1.119169


$$\begin{array}{c} Score(i,t)=x_{i}+y_{t} \end{array}$$ (2)

The keyword pair “child+maxchan (1st)”, which appeared in both Combined Models 1 and 2, indicates a structurally strong association between the two keywords. ‘child’ is a general target keyword related to CSAM within the dataset, and its combination with ‘maxchan’ highlights a network centered on this theme. These findings indicate that ‘maxchan’ functions as a central hub, connecting multiple keywords and CSAM-related sites rather than acting as an isolated platform. “lola+maxchan (2nd)” and “zoo+maxchan (3rd)” represent specific content and communities, respectively, and their combination with ‘maxchan’ confirms that these keyword pairs frequently co-occur within the network. These keyword pairs go beyond mere co-occurrence, implying that theme-specific crime-related terms are interconnected through ‘maxchan’ as a central hub. This structural pattern illustrates how networks form and materials are exchanged among child sexual abuse–related sites. The keyword pairs “myloveboard+maxchan (4th)” and “jblinks+maxchan (4th)” illustrate the flow of materials or navigation paths between platforms through categories or hyperlink structures. In particular, keywords like “jblinks” are not merely nodes within the network but also function as pathways facilitating access to other sites. The structural analysis of Combined Models 1 and 2 confirms that “maxchan” exerts substantial influence within the CSAM site network. The associated keyword pairs clarify the primary information flow and interconnections among nodes. This provides critical insights into the connection structure and material exchange mechanisms within the dark web network and highlights the role and importance of specific hub nodes within the network.

Combined Models 1 and 2 provided useful information for collecting crime leads, but several limitations were identified. First, the simple combination of eigenvector centrality and TF-IDF does not sufficiently ensure meaningful associations between keywords. For instance, “zoo+stronghold” was extracted as a key keyword pair by both methods, but the two keywords did not appear simultaneously on the same dark web site. This indicates that such keyword pairs may fail to represent a concrete relationship with crime leads. Second, since TF-IDF and eigenvector centrality rely on keyword frequency and importance, there is a possibility that frequently appearing or highly connected keywords may be evaluated as significant even if they are unrelated to actual crimes.

Overall, the analysis of the results from Combined Models 1 and 2 shows that the interpretation of keyword pairs remains limited to identifying frequently appearing keywords or tracking general crime trends across multiple CSAM-related sites. These limitations are attributed to the approach of simply combining top-ranked keywords from both methods to generate keyword pairs.

To address these issues and secure more reliable crime leads, we explore additional experiments by incorporating semantic similarity from Word2Vec into the eigenvector centrality method, as detailed in the following analysis.

#### Combined Models 3 and 4: Eigenvector centrality–Word2Vec based models.

This section presents the design of Combined Models 3 and 4, based on eigenvector centrality and Word2Vec. First, Combined Model 3 focuses on the top 20 critical criminal keywords identified through eigenvector centrality and utilizes Word2Vec to extract additional keywords. By combining the 20 keywords derived from eigenvector centrality with 120 unique keywords extracted from Word2Vec that do not overlap with eigenvector centrality, a total of 2,400 keyword pairs were generated. To maximize the reflection of the characteristics of the keywords extracted through the combined model, Equation (3) proposed in this study was applied to calculate the scores of the keyword pairs. In this context, the “characteristics of keywords” refer to the use of eigenvector centrality keywords as-is, while incorporating the values of related keywords with semantic similarity extracted based on the central eigenvector centrality keyword *i*.

Equation (3) was designed to calculate Score(i,j) by incorporating the characteristics of both eigenvector centrality and Word2Vec, enabling an evaluation of how effective each keyword pair is as a critical criminal lead.


$$\begin{array}{c} Score(i,j)=x_{i}+y_{j|i}ifi\in X,j\notin X \end{array}$$ (3)

Equation (3) applies when only keyword *i* belongs to the eigenvector centrality set *X*. Score(i,j) is calculated by accumulating the eigenvector centrality coefficient $x_{i}$ of *i* and the probability $y_{j|i}$ of *j* appearing centered around *i*. The higher the Score(i,j), the more effective the keyword pair for CSAM-related evidence collection. In this study, the priority of the keyword pairs is determined based on Score(i,j).

For example, if “lola” belongs to the eigenvector centrality set *X* and “sex” does not, the score for the keyword pair is calculated by considering both the eigenvector centrality value of ‘lola’ and the semantic similarity value of the related keyword “sex”, which is extracted using Word2Vec with “lola” as the central keyword. Unlike Combined Model 3, Combined Model 4 uses keywords extracted from eigenvector centrality as center keywords to further extract related keywords, and then reuses these newly extracted keywords as center keywords to extract additional keywords. This process results in the extraction of 1 to 94 related keywords for each eigenvector centrality keyword, generating a total of 1,886 keyword pairs In Combined Model 4, the Score(i,j) value is calculated using Equation (4), which, like Equation (3), is designed to fully reflect the characteristics of both eigenvector centrality and Word2Vec.


$$\begin{array}{c} Score(i,j)=x_{i}+x_{j}+max\left(y_{j|i},y_{i|j}\right)if(i\in X,j\in X)or(i\in X,j\notin X) \end{array}$$ (4)

Equation (4) considers two cases: when both keywords *i* and *j* belong to the eigenvector centrality set *X*, and when only *i* belongs to *X*. In both cases, the score is calculated using the eigenvector centrality values $x_{i}$ and $x_{j}$, and then adding the higher of the two Word2Vec similarity values: $y_{j|i}$ or $y_{i|j}$, along with the maximum of the two Word2Vec-based semantic similarity values: $y_{j|i}$ and $y_{i|j}$. In the first case, where both *i* and *j* belong to set X, the score is determined by summing the eigenvector centrality values of both keywords ($x_{i}$ and $x_{j}$), and adding the higher semantic similarity score between the two—*max*($y_{j|i}$, $y_{i|j}$). For example, if both “lola” and “child” are in set *X*, the final score includes their centrality values and the strongest semantic similarity between them as calculated by Word2Vec. In the second case, where only *i* belongs to *X* and *j* does not, the score is calculated by accumulating $x_{i}$ and *max*($y_{j|i}$, $y_{i|j}$).

Even though *j* is not structurally central, the model allows for its semantic relevance to be considered in combination with a central keyword. For example, if “city” is not in set *X* but “zoo” is, and “zoo” is set as *i*, then the score is calculated using $x_{i}$ and the higher of the two semantic similarity values between “zoo” and “city”. This unified scoring formula enables the model to reflect both structural importance and semantic closeness in the keyword pairing process.

The scores derived in this manner are used to extract the top 20 keyword pairs in Combined Model 4. By comparison, Combined Model 3 generated 2,400 keyword pairs based on non-overlapping keywords between eigenvector centrality and Word2Vec, and selected the top 20 pairs using Equation (3). In contrast, Combined Model 4 expands the semantic search space by iteratively retrieving additional keywords centered around the eigenvector keywords and evaluating them using Equation (4). Thus, Combined Model 3 generated 2,400 keyword pairs by combining 20 non-overlapping eigenvector centrality keywords with 120 semantically related terms from Word2Vec, and selected the top 20 pairs using Equation (3). In contrast, Combined Model 4 expanded this approach by recursively extracting related terms centered on eigenvector keywords and calculating their scores using Equation (4). The final top 20 keyword pairs identified through both models are summarized in Table 6.

**Table 6:** Top 20 keyword pairs extracted from combined models using eigenvector centrality and Word2Vec.

No	Combined Model 3		Score(i,j)	No	Combined Model 4		Score(i,j)
	Keyword Pairs				Keyword Pairs		
**1**	child	loves	1.997858	**1**	child	zoo	2.325762
**2**	child	models	1.997721	**2**	child	kitty	2.319952
**3**	child	alice	1.997663	**3**	lola	pedomoms	2.131775
**4**	child	library	1.99761	**4**	child	jblinks	2.130509
**5**	child	hard	1.997606	**5**	lola	myloveboard	2.099154
**6**	child	teen	1.997586	**6**	jblinks	zoo	2.065803
**7**	child	age	1.997552	**7**	child	pedomoms	2.058864
**8**	child	series	1.997493	**8**	pedomoms	zoo	1.997206
**9**	child	baby	1.997476	**9**	thumbnailed	toddlers	1.99478
**10**	child	policy	1.997451	**10**	myloveboard	pedomoms	1.986363
**11**	child	telegru	1.997445	**11**	lola	toddlers	1.986329
**12**	child	country	1.997444	**12**	pedomoms	toddlers	1.963294
**13**	child	material	1.997415	**13**	kitty	zoo	1.9393
**14**	child	needs	1.997364	**14**	child	thumbnailed	1.935475
**15**	child	guide	1.997351	**15**	myloveboard	toddlers	1.931028
**16**	child	face	1.997338	**16**	child	myloveboard	1.906389
**17**	child	secret	1.997332	**17**	pedomoms	thumbnailed	1.891138
**18**	child	thought	1.997312	**18**	lola	thumbnailed	1.886499
**19**	child	remember	1.997307	**19**	lola	zoo	1.884885
**20**	child	policies	1.997272	**20**	jblinks	pedomoms	1.864979

The analysis of keyword pairs in Combined Model 3 revealed that “child” emerged as the central keyword, highlighting its significance in eigenvector centrality analysis. Moreover, when combined with keywords extracted through the Word2Vec method related to CSAM, it suggests that “child” could serve as a critical clue in crimes involving children. Notably, keywords paired with “child” included “loves,” “models,” “alice,” “library,” and “hard.” While these individual keywords may seem superficially unrelated, the contextual analysis capabilities of Word2Vec indicate their potential to provide crucial insights into child sexual crimes. Among the top-ranking keyword pairs, “child+loves” (1st) serves as a significant clue indicating a connection to CSAM on the dark web. Meanwhile, “child+models” (2nd), although it might ostensibly refer to child models, may implicitly suggest sexual activities involving children in specific contexts. The keyword “alice” (3rd) was verified as the name of a child model recurrently linked to CSAM. Additionally, keywords such as “teen”, “age”, “baby” and “material” derived through Word2Vec may not appear directly as criminal terms. However, when paired with ‘child,’ they can be interpreted as crime-related clues referring to acts or materials linked to child sexual crimes.

In Combined Model 4, the analysis of keyword pairs identified “child+zoo” (1st), suggesting a connection on the dark web to child-animal sexual exploitation. Other high-ranking keyword pairs, such as “child+kitty” (2nd), “lola+pedomoms” (3rd), and “child+jblinks” (4th), were confirmed to be directly associated with CSAM, frequently appearing together on dark web sites related to such crimes. This indicates that these top-ranking keywords effectively reflect the significance of child sexual crime-related terms. However, lower-ranking keyword pairs, while appearing less relevant on the surface, may still carry latent connections to crimes due to their contextual pairing. This suggests the necessity of not excluding lower-ranking keyword pairs from analysis, as these may reveal seemingly less significant terms to be pivotal crime-related clues. Therefore, a comprehensive analysis using diverse techniques is essential to perform in-depth crime clue investigations and uncover additional leads. This section demonstrates that the integration of eigenvector centrality and the Word2Vec method enables the extraction of meaningful crime-related information beyond simple keyword combinations. The experiments with these Combined Models hold significant value in precisely identifying associations with crimes and expanding connections to potential criminal activities by securing additional keywords.

#### Comparative analysis of combined model performance.

**Fig 3:**
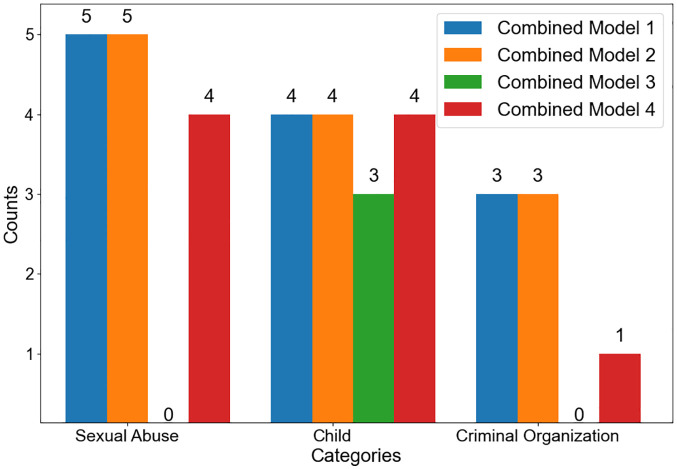
Categorization of the top 20 keywords extracted using combined text mining models by crime-related category.

We objectively evaluated the effectiveness of critical crime keyword collection by categorizing the keywords extracted through combined models 1, 2, 3, and 4 into categories such as sex crimes, children, and criminal organizations. Fig 3 shows the classification results of the top keywords extracted using the combined text mining methods.

Fig 3 illustrates that the top keywords from Combined Models 1 and 2 are grouped into five related to sexual crimes, five to children, and three to criminal organizations. The sexual crime category includes keywords such as “lola,” “pedomoms,” and “zoo.” The child category includes “child” and “toddlers,” while the criminal organization category includes “myloveboard” and “mixedlolitas.” In contrast, Combined Model 3 yielded no keywords related to sexual crimes or criminal organizations and only three related to children, namely “child,” “teen,” and “baby.” Compared to other models, this indicates that Combined Model 3 was less effective in collecting crime-related terms across categories. Finally, Combined Model 4 produced four sexual crime–related keywords, four child-related keywords, and one related to criminal organizations. All of these were validated as highly relevant to their respective categories.

The sexual crime category includes keywords such as “lola,” “pedomoms,” “zoo,” and “jblinks,” while the child category includes “child” and “toddlers.” The criminal organization category includes “myloveboard.” An analysis of the methods reveals that Combined Models 1 and 2, which utilized eigenvector centrality and TF-IDF, extracted a total of 19 keywords, forming keyword pairs. Among the extracted keywords, 12 from Combined Models 1 and 2 were confirmed to be valid across the categories of sexual crimes, children, and criminal organizations. In contrast, Combined Model 3 generated 21 keywords, yet only three were relevant to the child category.

This outcome suggests that, although Model 3 aimed to diversify the keyword pool by combining eigenvector centrality with non-overlapping keywords from Word2Vec, it was less effective in retrieving keywords directly related to the crime categories. Two primary factors may explain this result: (1) semantic discrepancies between the keyword sets derived from eigenvector centrality and Word2Vec, and (2) the exclusion of meaningful terms due to the strict emphasis on non-overlapping extraction.

On the other hand, Combined Model 4 produced only nine keywords—fewer than any other model—but all were confirmed to be valid within the key crime categories. Despite the limited number, this result highlights the model’s strength in generating high-relevance keywords by effectively capturing semantically associated terms through recursive keyword expansion. These findings support the potential of combining eigenvector centrality with TF-IDF and Word2Vec for generating effective keyword pairs that serve as crime leads in dark web investigations. Compared to using a single text-mining technique, this integrated approach enables broader keyword coverage and improved contextual relevance. In conclusion, eigenvector centrality consistently demonstrated superior performance in identifying core keywords tied to CSAM. Its ability to detect high-importance terms within the network structure makes it a particularly valuable technique for extracting direct and actionable CSAM-related investigative leads. Thus, a strategy centered on eigenvector centrality emerges as the most reliable and efficient method for analyzing dark web crime content.

#### Quantitative evaluation of model performance.

Each model’s effectiveness was quantitatively validated using search accuracy in the Torch dark web search engine. Top five keyword pairs from each model were used as queries. Table 7 presents the accuracy of these searches.

**Table 7:** Validation accuracy based on keyword pairs extracted from combined models.

Model	No	Keyword Pairs		Types of crime occurrences		Accuracy (%)
				Child sexual abuse material distribution	Sexual abuse Community	
				Number of Identified Sites (%)		
**Combined Models** **1 and 2**	1	child	maxchan	0 (0)	0 (0)	0
	2	lola	maxchan	0 (0)	0 (0)	0
	3	zoo	maxchan	0 (0)	0 (0)	0
	4	pedomoms	maxchan	0 (0)	0 (0)	0
	5	toddlers	maxchan	0 (0)	0 (0)	0
**Combined Model 3**	1	child	loves	373 (15.46)	1,765 (73.15)	88.61
	2	child	models	108 (8.96)	800 (66.39)	75.35
	3	child	alice	1,263 (87.22)	170 (11.74)	98.96
	4	child	library	7 (0.2)	3,207 (92.63)	92.83
	5	child	hard	107 (6.94)	1,071 (69.5)	76.44
**Combined Model 4**	1	child	zoo	1,259 (91.36)	90 (6.53)	97.89
	2	child	kitty	69 (10.69)	10 (73.4)	84.09
	3	lola	pedomoms	1,247 (99.84)	2 (0.16)	100
	4	child	jblinks	12 (100)	0 (0)	100
	5	lola	myloveboard	1,247 (100)	0 (0)	100

Since Combined Models 1 and 2 generated identical top 5 keyword pairs, they are presented together in a single row in Table 7 for clarity and conciseness. The analysis results showed that the keyword pairs extracted from Combined Models 1 and 2 — “child+maxchan” (1st, 0.00%), “lola+maxchan” (2nd, 0.00%), “zoo+maxchan” (3rd, 0.00%), “pedomoms+maxchan” (4th, 0.00%), and “toddlers+maxchan” (5th, 0.00%) — recorded no search results on the dark web search engine Torch. This demonstrates that Combined Models 1 and 2 are ineffective for identifying CSAM sites or collecting crime leads. These keyword pairs failed to reflect the actual search patterns or contexts used on the dark web, exposing limitations in contextual relevance and practical usability. Consequently, the practical applicability of these models is limited, and their effectiveness for searching and collecting data in the dark web environment is very low.

On the other hand, the top keyword pairs extracted from Combined Model 3 — “child+loves” (1st, 88.61%), “child+models” (2nd, 75.35%), “child+alice” (3rd, 95.96%), “child+library” (4th, 92.83%), and “child+hard” (5th, 76.44%) — achieved high accuracy. Notably, ‘child+loves’ and ‘child+library’ showed exceptional performance in detecting CSAM communities, serving as highly effective keywords for investigative lead collection. Combined Model 4 outperformed the other models, with top keyword pairs — “child+zoo” (1st, 97.89%), “child+kitty” (2nd, 84.09%), “lola+pedomoms” (3rd, 100.00%), “child+jblinks” (4th, 100.00%), and “lola+myloveboard” (5th, 100.00%) — showing consistently high accuracy in detecting child sexual exploitation content. These results demonstrate superior performance compared to the standalone eigenvector centrality model. Furthermore, Combined Model 4 significantly improved the efficiency of collecting CSAM sites and crime leads by accurately identifying a large number of relevant sites.

#### Robustness checks using multiple seed keywords.

In this part of the study, we conducted a multi-seed stability evaluation using pthc, upskirt, and pedomoms as seed keywords to minimize biases caused by relying on a single seed keyword and to assess the robustness of the model. These three keywords are among the most frequently used search terms for detecting CSAM on the dark web and commonly appear in CSAM-related posts and community discussions.

First, “pthc”, an abbreviation for “pre-teen hard core,” refers to illegal sexual exploitation material involving children under the age of 13 and is widely used as a high-risk tag on websites that share or trade such illicit content. “upskirt” is a term that indicates voyeuristic images or videos secretly taken under the skirt of a woman or minor. Lastly, “pedomoms” is a slang term derived from pedophile, referring to individuals who perceive their own children as sexual targets. Using these seed keywords, we collected text data with the dark web crawler developed in this study and evaluated the crime-site retrieval accuracy under the multi-seed setting. The results of this multi-seed evaluation for pthc, upskirt, and pedomoms are presented in Table 8.

**Table 8:** Multi-seed-based stability verification results for TF-IDF, eigenvector centrality, and Word2Vec models.

keyword	No	TF-IDF	Accuracy (%)	Eigenvector Centrality	Accuracy (%)	Word2Vec	Accuracy (%)
**pthc**	**1**	ivanivanov	0	incest	75.52	fotos	97.93
	**2**	maxchan	0	video	50.51	young	82.12
	**3**	admin	25	pedo	82.45	little	62.8
	**4**	terefere	0	rape	78.26	hole	72.66
	**5**	cooky	48.86	sex	58.83	nude	97.41
	**6**	stronghold	5.36	child	79.7	violence	78.97
	**7**	mixedlolitas	0	boys	90.74	taste	75.16
	**8**	language	77.66	teen	73.05	loli	68.61
	**9**	child	79.7	photo	61.12	sticky	18.8
	**10**	shorturl	0	porno	82.85	shorturl	0
**upskirt**	**1**	gallery	98.23	erotic	80.94	abuse	59.1
	**2**	erotic	80.94	gallery	98.23	plus	27.27
	**3**	upskirt	97.92	zenphoto	100	nikon	74.74
	**4**	zenphoto	100	image	78.08	country	73.02
	**5**	camera	85.43	upskirt	97.92	cock	70.07
	**6**	image	78.08	aperture	98.26	cooky	46.86
	**7**	country	73.02	shutter	95.97	iphone	22.95
	**8**	aperture	98.26	compensation	93.77	baby	81.7
	**9**	shutter	95.97	model	70.36	corporation	59.09
	**10**	compensation	93.77	camera	85.43	amaliiaa	0
**pedomoms**	**1**	maxchan	0	child	79.7	sex	58.83
	**2**	mixedlolitas	0	pedomoms	97.54	school	62.3
	**3**	child	79.7	zoo	88.68	boys	90.74
	**4**	stronghold	5.36	kitty	62.63	loli	68.61
	**5**	boys	90.74	toddler	92.63	young	82.12
	**6**	shorturl	0	myloveboard	100	abuse	59.1
	**7**	pool	33.5	thumbnailed	92.99	porno	82.85
	**8**	kitty	62.63	smoothly	24.51	photo	61.12
	**9**	society	90.1	community	43.56	age	81.03
	**10**	illegal	31.19	monero	78.81	dick	78.17

Table 8 presents a comparison of the top 10 extracted keywords for each seed keyword—“pthc”, “upskirt”, and “pedomoms”—based on their accuracy in identifying CSAM-related dark web sites. The analysis evaluates the performance of “TF–IDF”, “Eigenvector Centrality”, and “Word2Vec”, focusing specifically on the core crime-related keywords extracted by each method. For the seed keyword “pthc”, the “TF–IDF” method yielded a substantial number of keywords with 0% or very low accuracy, with only “child” (79.7%) and “language” (77.66%) showing relatively high accuracy. This indicates a limitation in “TF–IDF”’s ability to extract high-quality core CSAM-related keywords. In contrast, the “Eigenvector Centrality” method produced several high-accuracy keywords such as “boys” (90.74%), “pedo” (82.45%), and “child” (79.7%). Among these, “boys” and “child” directly reference offenses against minors, whereas “pedo” and “incest” denote sexual abuse and taboo relations—terms frequently used within actual CSAM communities. “Word2Vec”, meanwhile, extracted keywords such as “fotos” (97.93%), “nude” (97.41%), and “young” (82.12%), which are strongly associated with visual or age-related cues. However, unrelated terms such as “sticky” (18.8%) and “shorturl” (0%) were also included, revealing limitations in selectively isolating core crime indicators during contextual expansion.

For the seed keyword “upskirt”, the “TF–IDF” method produced highly accurate technical terms related to illicit recording and gallery management—”zenphoto” (100%), “gallery” (98.23%), and “aperture” (98.26%). “Eigenvector Centrality” similarly extracted “zenphoto” (100%), “upskirt” (97.92%), and “gallery” (98.23%), demonstrating strong overlap with “TF–IDF” and presenting consistently high accuracy, thereby confirming their suitability as core CSAM-related keywords. In contrast, “Word2Vec” returned general or camera-related terms such as “nikon” (74.74%), “country” (73.02%), and “cock” (70.07%), while many remaining keywords showed lower accuracy, indicating limited effectiveness in pinpointing core crime indicators for this seed.

For the seed keyword “pedomoms”, the “TF–IDF” method produced a few high-accuracy terms such as “boys” (90.74%), “society” (90.1%), and “child” (79.7%), whereas most other keywords showed 0% or low accuracy, demonstrating restricted capability in identifying core CSAM-related terms. Conversely, “Eigenvector Centrality” extracted “pedomoms” (97.54%), “myloveboard” (100%)—a known board or community name used for sharing illegal child content—and “toddler” (92.63%), a term denoting very young children. These keywords are widely observed in real CSAM contexts, supporting the method’s ability to reliably capture crime-specific terminology. “Word2Vec” extracted partially relevant terms such as “boys” (90.74%), “school” (62.3%), and “abuse” (59.1%), but its overall accuracy remained lower than that of “Eigenvector Centrality”. Moreover, several extracted terms (e.g., “school”) are commonly used in non-criminal, everyday contexts, indicating a tendency to include keywords whose meaning may become ambiguous when interpreted within a crime-specific environment.

Overall, across all three seed keywords—“pthc”, “upskirt”, and “pedomoms”—”Eigenvector Centrality” consistently achieved the highest average accuracy and exhibited stable structural coherence. While “TF–IDF” and “Word2Vec” captured certain core CSAM-related terms through document-based weighting or semantic expansion, they frequently introduced unrelated keywords or showed reduced accuracy in identifying crime-specific sites. In contrast, “Eigenvector Centrality” reliably extracted core crime-related keywords directly associated with CSAM across all seed configurations. These findings empirically demonstrate that even without relying solely on a single seed keyword such as “lolita”, the eigenvector-based approach can robustly and consistently identify key CSAM-related terminology in diverse seed keyword environments.

## Discussion

The results of the multi-seed robustness analysis confirm that the proposed framework does not depend on a single seed keyword, such as “lolita,” and can consistently identify core child sexual abuse material (CSAM)–related terminology across heterogeneous seed environments using different seed keywords (pthc, upskirt, and pedomoms). In particular, the eigenvector centrality–based approach exhibited stable performance across all evaluated seed keywords, indicating its effectiveness in capturing structurally meaningful crime-related terms that are deeply embedded within CSAM-related communities. This robustness is especially important in real-world investigative settings, where initial seed keywords may vary depending on case types, platforms, or newly emerging slang. Unlike conventional approaches that rely solely on document-level frequency or simple semantic similarity, the proposed framework effectively integrates complementary analytical techniques, thereby providing a robust and scalable keyword expansion mechanism that remains applicable in the rapidly evolving dark web environment.

Despite these strengths, the proposed framework has a limitation. Because both the corpus construction and the subsequent validation processes in this study were conducted based on content accessible through the Torch search engine, the analytical results may not fully reflect the diversity of CSAM-related content across the entire dark web, but may instead primarily capture characteristics of data observed within the Torch-accessible ecosystem. Such dependency may influence the interpretation of retrieval performance under certain environments or conditions. Nevertheless, Torch is currently one of the most accessible dark web search engines and provides a practical and appropriate data foundation aligned with the scope and objectives of this study.

In addition, the search-hit–based accuracy metric used in this study was calculated using only non-duplicated search results for each seed keyword, thereby limiting potential distortions caused by duplicate pages. However, depending on the characteristics of the initial seed keywords selected for collecting search results, factors such as term ambiguity, search engine ranking bias, and top-k cutoff settings may partially affect the measured accuracy, leading to a potential overestimation of performance. To account for this possibility, we conducted an additional multi-seed robustness analysis rather than relying on a single seed keyword. The results demonstrated consistent performance trends across different seed environments, thereby jointly validating the stability and reliability of the observed findings.

## Conclusion

As crimes on the dark web become increasingly diverse and sophisticated, investigators must devote substantial time and effort to understanding criminal activity across platforms. In particular, child sexual abuse is severely punished worldwide, and viewing CSAM increases the risk of further abuse by fostering the perception of children as sexual objects. Therefore, developing technologies that can assist in the detection and investigation of such crimes is crucial.

To address this challenge, we conducted a text-mining-based investigation aimed at systematically collecting investigative clues related to child sexual abuse crimes on the dark web. To achieve this, we developed a custom crawler that collected text data from 2,414 dark web pages, resulting in a dataset of approximately 71,666 nouns. Using this dataset, we applied three text mining techniques—TF-IDF, Eigenvector Centrality, and Word2Vec—to extract the top 20 keywords with high structural or semantic significance. Among the individual models, Eigenvector Centrality proved to be the most effective for identifying CSAM-related keywords. Building on these results, we designed combined models that integrated Eigenvector Centrality with TF-IDF or Word2Vec. Combined Model 4, which incorporated Word2Vec-based semantic similarity into the Eigenvector-based keyword set, achieved superior retrieval accuracy ranging from approximately 84% to 100%, clearly outperforming the other approaches. This demonstrates the value of combining structural and semantic methods, with Eigenvector Centrality serving as a robust foundation. Importantly, the proposed methodology enables the dynamic expansion of early-stage investigative clues by automatically generating semantically associated keywords, moving beyond static keyword sets. This allows investigators to adapt more effectively to evolving dark web threats and content variations. In future work, we aim to extend the proposed crime information collection model to track dark web activities linked to social media platforms such as Telegram and Discord, ultimately evolving it into an integrated investigative support framework capable of operating across diverse platforms. Within this expanded system, the keyword-ranking–based clue identification framework developed in this study can function as a core module, automatically filtering and prioritizing large-scale dark web data to substantially reduce the volume of information investigators must manually review. This will further enable the system to directly support investigative workflows by identifying and monitoring high-risk sites that should be examined with priority.
